# Efficacy of ceftazidime-avibactam for the treatment of central nervous system infection caused by carbapenem-resistant *Klebsiella pneumoniae* after neurosurgery

**DOI:** 10.3389/fneur.2025.1604045

**Published:** 2025-10-22

**Authors:** Wenkai Cong, Guanghong Qi, Shengjie Wang, Zhenwei Bai, Lin Wang, Hongwei Chen

**Affiliations:** ^1^Department of Cerebrospinal Fluid Disease Neurosurgery, Aviation General Hospital, Beijing, China; ^2^Department of Neurosurgery, Aviation General Hospital, Beijing, China

**Keywords:** carbapenem-resistant *Klebsiella pneumoniae*, central nervous system infection, ceftazidime-avibactam, polymyxin, tigecycline

## Abstract

**Background:**

Carbapenem-resistant *Klebsiella pneumoniae* (CRKP) infections of the central nervous system (CNS) are associated with high mortality rates. These infections are particularly challenging to treat due to both bacterial resistance and the protective blood–brain barrier. This study aims to evaluate treatment outcomes for CRKP-induced CNS infections and provide insights into effective therapeutic strategies for similar cases.

**Methods:**

A retrospective analysis was conducted on patients with CRKP-positive cerebrospinal fluid (CSF) samples admitted to the CSF Neurosurgery Department of the Aviation General Hospital between August 2019 and November 2021. Data collected included patient demographics, medical history, laboratory results, etiological findings, and antibiotic treatments. Nine patients with CRKP-induced CNS infections who were treated with Ceftazidime-avibactam (CAZ-AVI) were included in the analysis. The study assessed clinical features, treatment courses, and patient outcomes.

**Results:**

Of the nine patients, 88.9% (8/9) achieved both clinical and etiological cure. One patient experienced relapse 20 days after initial CSF culture negativity, with the family opting for discontinuation of further treatment. Following infection resolution, patients exhibited varying degrees of neurological improvement, including one case of complete recovery from a comatose state (GCS 6 to GCS 15). Treatment-related adverse effects included transient liver dysfunction in one patient and diarrhea in three, which resolved with symptomatic management. CSF drainage tubes were removed in three patients following treatment, while five required shunt surgery due to hydrocephalus. No relapses were reported in treated patients during a follow-up period of 3–12 months.

**Conclusion:**

Intravenous CAZ-AVI combined with intracerebroventricular or intrathecal polymyxin B or amikacin demonstrated promising efficacy as a treatment regimen for CRKP-induced CNS infections.

## Introduction

Central nervous system (CNS) infections caused by carbapenem-resistant *Klebsiella pneumoniae* (CRKP) have emerged as a significant and life-threatening public health concern, owing to their increasing prevalence and associated mortality (1). The selection of appropriate antibiotics is crucial for optimizing treatment outcomes in these infections. However, the therapeutic options are severely limited due to the challenges posed by the blood–brain barrier and the growing prevalence of bacterial drug resistance (2). Ceftazidime-avibactam (CAZ/AVI) is a combination of the third-generation cephalosporin ceftazidime and the synthetic *β*-lactamase inhibitor avibactam, which has been demonstrated to be effective in treating complicated urinary tract infections, intra-abdominal infections, and hospital-acquired pneumonia caused by Gram-negative bacilli (3). The growing body of evidence supporting the efficacy of CAZ/AVI in treating CRKP-induced CNS infections is reflected in several case reports (2–6), particularly those by Qing et al. (5) and Zhang et al. (6), further demonstrating the potential of CAZ/AVI in managing such infections. In this study, medical records were reviewed for 9 patients with CRKP-induced CNS infections who were treated with CAZ/AVI at the CSF Neurosurgery Department of the Aviation General Hospital. Clinical features, diagnostic and treatment protocols, patient outcomes, and prognosis were analyzed to provide guidance for the management of similar cases. This study presented a case series involving a small cohort of 9 patients, demonstrating the limited availability of data on the management of CRKP-induced CNS infections. While the treatment protocol described has shown promising results, it is crucial to interpret these findings in the context of the limited sample size and the retrospective design of the study. Furthermore, a comprehensive review of the existing literature on this topic has been included to contextualize the findings of this case series within the broader scope of available treatments for similar infections.

## Objectives

This investigation aimed to assess the effectiveness of CAZ/AVI in treating CRKP-induced CNS infections following neurosurgery.

## Materials and methods

### General information

This retrospective study included 9 patients who were diagnosed with CRKP-induced CNS infections, confirmed by positive cerebrospinal fluid (CSF) cultures between August 2019 and November 2021 at the CSF Neurosurgery Department of the Aviation General Hospital. Initially, a total of 15 patients who tested positive for CRKP in CSF cultures were reviewed. After excluding those who did not meet the inclusion criteria, such as patients with incomplete medical records or those who did not receive CAZ-AVI therapy, a final cohort of 9 patients was included in the study (Table 1). The selection of patients was based on their CRKP-positive CSF cultures and the subsequent treatment with CAZ/AVI. Data were extracted from medical records, including patient demographics (age, sex), primary disease, neurosurgical history, and prior antibiotic treatments.

The cohort comprised 8 men and 1 woman aged 15 to 61 years. The primary conditions included cerebral hemorrhage in 3 patients, traumatic brain injury in 2, brain abscess in 2, intracranial space-occupying lesion in 1, and toxic encephalopathy in 1. All patients had a history of neurosurgery and had received multiple antibiotic courses prior to CRKP infection. At the time of presentation, 6 patients were comatose, 2 had blurred consciousness, and 1 was lethargic. All patients exhibited fever (body temperature >38 °C) and positive signs of meningeal irritation. Toxic encephalopathy is a reversible brain dysfunction caused by toxic substances, such as drugs, infections, or metabolic disturbances. In this case, the patient had a history of exposure to neurotoxic agents, which contributed to altered mental status and neurological impairment, alongside other clinical manifestations of CRKP infection.

**Table 1:** Results of CSF cultures and *in vitro* susceptibility testing of antibiotics in 9 patients.

Patient number	Results of CSF cultures	Results of *in vitro* susceptibility testing of antibiotics						
		CAZ/AVI	COL	TGC	AMK	MNO	MEM	SCF
1	CRKP	S	S	S	S	R	R	R
2	CRKP	S	S	S	R	R	R	R
3	CRKP	S	S	S	R	R	R	R
4	CRKP	S	S	S	R	S	R	R
5	CRKP	S	S	S	R	R	R	R
6	CRKP	S	S	S	S	R	R	R
7	CRKP	S	S	S	S	R	R	R
8	CRKP	S	S	S	S	R	R	R
9	CRKP	S	S	S	S	R	R	R

CAZ/AVI, Ceftazidime-avibactam; COL, Colistin; AMK, Amikacin; TGC, Tigecycline; MNO, Minocycline; S, Sensitivity; R, Resistance; CRKP, Carbapenem-resistant; SCF, Cefoperazone/Sulbactam; MEM, Meropenem.

The study was approved by the Medical Ethics Committee of the Aviation General Hospital (HK2022-08). Due to its retrospective nature and anonymized data collection, the requirement for informed consent was waived in accordance with ethical guidelines. The study adhered to the principles outlined in the Declaration of Helsinki (revised 2013).

### Treatment

Upon hospital admission, intracranial implants were removed, or craniotomy was performed to clear abscesses within 72 h of admission. Ventricular/lumbar cistern drainage was performed in 7 patients, while 2 patients retained their original ventricular drainage devices. All patients received a combination of intravenous and intracerebroventricular/intrathecal antibiotics, based on drug susceptibility testing. Further details of antibiotics and dosages are presented in Table 2. The treatment regimen was tailored to the individual resistance profiles of the CRKP strains identified. Dosages of intrathecal antibiotics, including CAZ/AVI, were tailored according to the patient’s renal function, assessed via estimated glomerular filtration rate (eGFR). In cases where CAZ/AVI was used, the dose was adjusted for individual patients, ensuring optimal therapeutic levels. Additionally, the minimum inhibitory concentration (MIC) of CAZ/AVI against CRKP strains was determined for each patient using standard broth microdilution methods. The MIC values were interpreted according to the Clinical and Laboratory Standards Institute (CLSI) guidelines, with results categorized as susceptible, intermediate, or resistant. The PK/PD (pharmacokinetics/pharmacodynamics) targets for CAZ/AVI therapy were adjusted based on these MIC values to ensure optimal drug exposure and target attainment. For each patient, the probability of target attainment (PTA) was calculated based on the MIC and the recommended dosing regimen to assess the likelihood of effective treatment. This adjustment was crucial in determining the adequacy of CAZ/AVI therapy, particularly given the varying resistance profiles of CRKP strains in CNS infections. Intravenous colistin was not administered to patients in this study due to concerns about its potential systemic toxicity, particularly in the setting of renal impairment and critical illness. Instead, intrathecal colistin was selected to achieve high local concentrations in the CSF, directly targeting the infection site while minimizing systemic side effects. The decision to use intrathecal colistin in combination with CAZ/AVI was based on the principle of optimizing treatment efficacy at the site of infection, particularly for CRKP strains, which are often resistant to multiple antibiotics. The use of intrathecal colistin also allowed for a more targeted therapeutic approach, potentially reducing the risk of adverse systemic effects, which are a concern with intravenous administration.

**Table 2:** Clinical features, diagnosis and treatment process, and outcomes of 9 patients.

Patient number	Sex	Age	Primary disease	History of neurosurgery	History of antibiotics use	CSF drainage or shunt device	Antibiotics used and course of treatment	Antibiotic side effects	Time to CSF culture turning negative	Cerebrospinal fluid test results at different time points				Result	State of Consciousness	
										Glucose (mmol/L)		Protein (g/L)			On admission	Cure of infection
										Diagnosis	Cure	Diagnosis	Cure			
1	Male	56	Intracranial space-occupying lesion	Craniotomy for tumor resection	Etimicin, piperacillin, CEPs	Drainage tube in the operation area	Intravenous CAZ/AVI for 18 days, TGC for 7 days; intraventricular AMK for 5 days	Diarrhea	Day 10 of medication	0.90	2.56	2.84	0.44	Cured, without relapse during the 6-month follow-up	Confusion (GCS-12)	Full conscious (GCS-15)
2	Male	15	Intracranial aneurysm rupture and hemorrhage	DC, EVD, LD, Ommaya sac implantation	CEPs, MNO	VP, Ommaya sac	Intravenous TGC and PB for 35 days, intraventricular PB for 35 days, still positive for CSF cultures Shift to intravenous CAZ/AVI for 39 days, and intraventricular PB for 8 days	No	Day 42 of medication	0.05	3.61	3.34	0.24	Cured, without relapse during the 12-month follow-up	Coma (GCS-6 T)	Full conscious (GCS-15)
3	Male	33	Toxic encephalopathy	VP, with VP removed, then VP again, LD	CEPs, PB	VP, LD	Intravenous CAZ/AVI for 46 days, PB for 24 days; intraventricular PB for 12 days	No	Day 10 of medication	0.02	3.22	10.66	0.37	Cured without relapse during the 3-month follow-up	Coma (GCS-5 T)	Coma (GCS-7 T)
4	Female	61	Brain abscess	Abscess removal by craniotomy	CEPs, VA	Drainage tube in the operation area	Intravenous TGC for 17 days, CAZ/AVI for 17 days; intraventricular PB for 17 days	No	Day 14 of medication	2.17	4.22	1.12	0.09	Cured, without relapse during the 6-month follow-up	Coma (GCS-7 T)	Confusion (GCS-10)
5	Male	21	Traumatic brain injury	DC, VP, VP removed, EVD	CEPs, TGC	EVD	Intravenous TGC for 39 days, CAZ/AVI for 14 days; intraventricular PB for 13 days	No	Day 5 of medication	1.74	4.36	–	–	The infection relapsed after turning negative for CSF cultures for 20 days, and the patient gave up treatment.	Coma (GCS-6 T)	–
6	Male	42	Intracranial aneurysm rupture and hemorrhage	Interventional embolization of aneurysm, EVD, DC, LD, VP, VP removed	CEPs, TGC	EVD, LD	Intravenous TGC for 12 days, CAZ/AVI for 49 days; intraventricular PB for 12 days	Diarrhea	Day 5 of medication	0.26	4.50	3.98	0.19	Cured, without relapse during the 6-month follow-up	Coma (GCS-7 T)	Confusion (GCS-10)
7	Male	33	Rupture and hemorrhage of cerebrovascular malformations	Interventional embolism, DC, EVD	CEPs, MEM, TGC	EVD	Intravenous TGC for 5 days, CAZ/AVI for 21 days; intraventricular PB for 10 days	ALT ↑	Day 8 of medication	0.58	4.04	3.77	0.21	Cured, without relapse during the 3-month follow-up	Coma (GCS-5 T)	Coma (GCS-7 T)
8	Male	15	Brain abscess	Abscess removal by craniotomy, LD	VA, MEM	LD	Intravenous CAZ/AVI for 46 days, intraventricular PB for 15 days	No	Day 14 of medication	0.08	2.84	2.48	0.51	Cured, without relapse during the 3-month follow-up	Drowsiness (GCS-14)	Full conscious (GCS-15)
9	Male	47	Traumatic brain injury	DC, LD, EVD, neuroendoscopy	CEPs, AMK, TGC	EVD	Intravenous CAZ/AVI for 32 days, intraventricular PB for 9 days	Diarrhea	Day 3 of medication	1.68	2.85	2.25	0.48	Cured, without relapse during the 3-month follow-up	Confusion (GCS-12)	Full conscious (GCS-15)

EVD, external ventricular drainage; VP, ventriculoperitoneal; LD, lumbar drainage; CRKP, Carbapenem-resistant *Klebsiella pneumonia*e; DC, decompressive craniectomy; CAZ/AVI, Ceftazidime–avibactam; COL, Colistin; AMK, Amikacin; TGC, Tigecycline; MNO, Minocycline; CEPs, Cephalosporins; VA, Vancomycin; MEM, Meropenem; ALT, alanine aminotransferase.

For patients with hydrocephalus, ventriculoperitoneal (VP) shunt surgery was performed after infection resolution. CSF drainage tubes were removed in patients who did not develop hydrocephalus.

### Vital signs and laboratory findings

Symptom changes and vital signs were monitored before, during, and after treatment. CSF samples were collected weekly for routine analysis, including biochemistry tests and bacteriological cultures to assess the bacterial load and progress. In addition, routine blood tests, as well as liver and kidney function tests, were performed at regular intervals. Electrolyte level was also monitored throughout the treatment course to detect any imbalances associated with antibiotic therapy.

### Efficacy evaluation

The primary outcome was the cure rate, defined as both etiological cure and clinical cure. Etiological cure was defined as the absence of CRKP in three consecutive CSF cultures after the initiation of treatment. Clinical cure was defined by the following criteria:

1. Normal body temperature for three consecutive days.
2. Improvement or resolution of original clinical symptoms (e.g., signs of meningeal irritation).
3. CSF glucose level exceeding 2.2 mmol/L in three consecutive tests, and a leukocyte-to-erythrocyte ratio below 1:500.

Patients were considered cured if both etiological cure and clinical cure were achieved. The time to treatment response and any relapses were also recorded.

### Safety assessment

Adverse reactions associated with the antibiotic regimen were closely monitored, with special attention to potential hepatic and renal toxicity, as well as electrolyte disturbances, hematological changes (e.g., anemia, leukopenia, thrombocytopenia), and gastrointestinal side effects (e.g., nausea, vomiting, and diarrhea). The incidence of each adverse reaction was documented, and the severity of adverse events was graded according to standard clinical protocols. The study also assessed whether any severe adverse effects led to treatment modification or discontinuation.

### Statistical analysis

Descriptive statistics were employed to analyze patient characteristics and outcomes. Continuous variables (e.g., age, treatment duration) were reported as median and range. Categorical variables (e.g., sex, primary disease type) were presented as frequencies and percentages. Efficacy outcomes (e.g., cure rate, time to response) were also analyzed descriptively. Data analysis was conducted using Excel 16.0 software (Microsoft Corporation, United States).

## Results

### Clinical efficacy

The cure rate (both etiological cure and clinical cure) was 88.9% (8/9). One patient relapsed 20 days after turning negative for CSF bacterial cultures. The relatives of this patient later gave up the treatment. Upon the diagnosis, the CSF protein level (range, 1.12–10.66 g/L) was increased, and the CSF glucose level (range, 0.02–2.17 mmol/L) was decreased in all patients. The CSF glucose and protein levels fell within normal ranges when the infection was cured (Table 2). The patients’ consciousness was improved to varying degrees after the infection was cured. Return to consciousness (GCS15) from a coma (GCS-6 T) was the best improvement. CSF drainage tubes were removed in 3 patients after the infection was cured. Shunt surgery was performed on the remaining five patients due to hydrocephalus.

### Safety assessment

Abnormal liver functions were found in one patient; 3 patients developed diarrhea, which was mitigated after symptomatic treatment.

### Long-term follow-up

All cured patients were followed up for 3–12 months (average, 5.3 months) after discharge. The infection or hydrocephalus did not recur in any of these patients.

## Discussion

The detection rate of *Klebsiella pneumoniae* and *Acinetobacter baumannii*, both typical Gram-negative pathogens, has risen significantly in recent years due to the widespread use of antibiotics. In China, there has been a notable increase in the detection of CRKP in CSF. The resistance rate to meropenem has escalated from 13.1 to 30.9%, while resistance to imipenem has increased from 12.6 to 30.4% (7). The CDC’s “Antibiotic Resistance Threats in the United States” report demonstrats bacterial drug resistance as a major threat, particularly among hospitalized patients (8). Due to the high drug resistance of CRKP, only a limited number of antibiotics remain effective for the treatment of CRKP-induced infections, including polymyxin, tigecycline, aminoglycosides, and CAZ-AVI (1, 9, 10). In this study, the observed cure rate of 88.9% with the combination of intravenous CAZ/AVI and intracerebroventricular/intrathecal polymyxin or amikacin supports the increasing recognition of CAZ/AVI as an effective therapeutic option for CRKP-induced CNS infections. This is particularly significant in light of its capacity to penetrate the blood–brain barrier and its demonstrated efficacy against KPC and OXA-48 enzymes (11–13). These findings are in line with previous case reports showing the efficacy of CAZ/AVI in treating CRKP infections (14, 15), while the present study expands this evidence to a broader patient population. Given the high variability in pharmacokinetics and the potential for altered drug penetration in the CNS, the dose of CAZ/AVI and other antibiotics should be carefully considered. In this study, the MIC of CAZ/AVI against CRKP strains was measured for each patient, and the drug dosages were adjusted accordingly to ensure efficacy. This was important as some strains exhibited intermediate MIC values, demonstrating partial susceptibility or the potential for resistance development if the dosage was inadequate. The results indicate that monitoring MIC is crucial for determining the effectiveness of CAZ/AVI in treating CRKP-induced CNS infections. The inclusion of MIC data may further strengthen our understanding of the treatment outcomes and help tailor therapy more effectively for individual patients.

The concentration of tigecycline in CSF is typically low, and its efficacy for CNS infections remains controversial (16). While some reports have described the successful treatment of CRKP-induced CNS infections with intravenous and intraventricular tigecycline, such cases remain rare (1, 17). Furthermore, intraventricular tigecycline is not officially approved for use in this context. Both polymyxin B/E and aminoglycosides have limited ability to penetrate the blood–brain barrier (14). Guidelines recommend the combined local administration of these antibiotics when intravenous therapy proves ineffective (18). A retrospective study conducted by our center in 2020 reported successful treatment of CRAB- or CRKP-related CNS infections with a combination of intravenous and intraventricular polymyxin B, with no significant side effects observed (16). In the present study, 7 patients received intraventricular/intrathecal polymyxin B, and one patient was treated with intraventricular amikacin. All patients achieved resolution of the infection without evidence of nephrotoxicity or neurotoxicity. These results suggest that intraventricular/intrathecal polymyxin B or amikacin may serve as an adjunctive treatment for CRKP-induced CNS infections.

However, there is a paucity of clinical studies evaluating the efficacy of CAZ/AVI for the treatment of CNS infections, with the majority of available literature consisting primarily of case reports. Both CAZ and AVI have been reported to exhibit high CSF penetration, which is crucial for the effective treatment of CNS infections (18). In a rabbit model, CAZ and AVI demonstrated 38% CSF penetration and reduced CSF bacterial loads by an average of 5.66 log10 cfu over an 8-h period (19). Yasmin documented a case in which CAZ/AVI, in combination with amikacin, was used to treat CRKP-induced CNS infections following surgery. With an 8-h dosing interval, the CSF concentration of CAZ was found to be 4 to 5 times higher than the MIC, while the CSF concentration of AVI remained above 1–2.5 mg/L for 50% of the dosing interval (20). According to these findings, we administered intravenous CAZ/AVI (2.5 g every 8 h) in combination with other sensitive antibiotics, either intravenously or via local administration. The overall cure rate in our cohort was 88.9% (8/9). In contrast, a regression analysis conducted by Toun et al. indicated that the attributable mortality rate for CRKP-induced CNS infections exceeded 50% (21). This discrepancy may be attributable to the relatively small sample size in their study; however, it also suggests that CAZ/AVI may represent a reasonable therapeutic option for CRKP-induced CNS infections. Nevertheless, further research is required to corroborate these findings. Additionally, some patients in the present study experienced mild adverse effects, including hepatic dysfunction and diarrhea, both of which resolved with symptomatic treatment. Despite the mild nature of these adverse reactions, it is imperative that close monitoring be conducted during treatment.

In a study conducted by Samuel et al. (22), intravenous CAZ/AVI alone could successfully treat CRKP-induced meningitis following neurosurgery. However, in the present study, CAZ/AVI was combined with either intravenous or local administration of other sensitive antibiotics, as the CSF concentration of CAZ/AVI was not monitored, and it was uncertain whether the drug achieved a therapeutically effective concentration in the CSF. To address this limitation, collaboration with an external research institution is being sought for further investigation. Additionally, a separate study demonstrated that CAZ/AVI, in combination with fosfomycin, ertapenem, and tigecycline, reduced the MIC of CRKP below the critical threshold, exhibiting a significant synergistic effect (23). Furthermore, another study evaluating combined antibiotic therapy for CRKP found that the combination of CAZ/AVI with amikacin, aztreonam, polymyxin, or fosfomycin reduced the MIC for *Klebsiella pneumoniae* by a factor of four (24). Gatti et al. (25) presented two cases of post-neurosurgical ventriculitis caused by carbapenem-resistant Gram-negative pathogens, successfully treated with high-dose CAZ/AVI. They emphasized the importance of a real-time clinical pharmacological advice program, which enabled optimization of PK/PD targets over time at the infection site, ultimately facilitating the successful treatment of these infections. This study suggests that high-dose CAZ/AVI-based regimens, when used in conjunction with other antibiotics, may offer a promising therapeutic strategy for managing refractory CRKP-induced infections. However, further validation through larger clinical trials is necessary to confirm the anti-infective efficacy of this regimen. In terms of pharmacokinetics, several recent studies have demonstrated the importance of CAZ/AVI’s CNS penetration, especially in the context of infections caused by multidrug-resistant pathogens. For instance, Xu et al. (26) demonstrated that CAZ/AVI exhibits adequate pharmacokinetic and pharmacodynamic properties for treating central nervous system infections caused by carbapenem-resistant Gram-negatives. Their findings demonstrate the potential of CAZ/AVI in penetrating the blood–brain barrier, which is crucial for treating CNS infections like ventriculitis (26). Additionally, Chen et al. (27) reported on pharmacodynamic target attainment at the infection site during treatment of post-neurosurgical ventriculitis caused by carbapenem-resistant *Klebsiella pneumoniae*. Their case study demonstrated that CAZ/AVI, when employed as part of a multi-drug regimen, successfully achieved the desired pharmacodynamic targets, thus providing a robust basis for its use in treating CNS infections. These findings, in conjunction with Gatti et al.’s results (25), confirm the therapeutic potential of CAZ/AVI for CNS infections caused by multidrug-resistant pathogens, while further clinical studies are needed to establish its efficacy and safety in this challenging clinical context. Given the limited therapeutic options available for CRKP-induced CNS infections, we emphasize the necessity of fostering enhanced collaboration with the laboratory medicine department. Additionally, *in vitro* susceptibility testing should be routinely performed to guide the selection of the most appropriate antibiotics for each individual case.

The Infectious Diseases Society of America (IDSA) guidelines recommend over 4 weeks of treatment for CRKP-induced meningitis. However, the specific treatment duration should be determined according to the severity of the infection and the clinical response of patients (14). In the present study, antibiotics were discontinued when the CNS infection-related symptoms and signs disappeared and the CSF cultures (negative for at least three consecutive CSF cultures, with an interval of over 24 h) and the radiographic findings were normal. Apart from antibiotic use, it was equally important to remove the intracranial implants if the patients were infected (14). Given the blood–brain barrier’s limitations in treating CNS infections, local antibiotic administration, whether intraventricular or intrathecal, has attracted attention as an adjunct to intravenous therapy. The success of intraventricular polymyxin B and amikacin in the present study is consistent with previous reports, where these antibiotics were used to treat infections that did not respond to systemic therapy (16, 24). Despite the promising results, only 7 out of 9 patients in this cohort received intrathecal or intraventricular antibiotics, demonstrating a potential gap in treatment optimization. The absence of serious neurotoxicities or kidney impairment in our cohort suggests that these treatments are safe when closely monitored.

While the efficacy of CAZ/AVI in treating CNS infections has been explored in case reports, this study provided new insights by assessing a broader patient population and incorporating detailed pharmacodynamic data, such as MIC measurements and dosing adjustments. These findings strengthen the growing body of evidence supporting CAZ/AVI as an effective treatment option for CRKP-induced CNS infections, particularly given the drug’s ability to penetrate the blood–brain barrier and its action against KPC and OXA-48-producing pathogens. However, Zhao et al. (28) previously demonstrated the effectiveness of CAZ/AVI-based combination therapy for hospital-acquired CNS infections caused by carbapenem-resistant *Klebsiella pneumoniae*, aligning with the high cure rates found in patients treated with CAZ/AVI in combination with other antibiotics. Zhao et al.’s study (28) provided valuable data on the clinical outcomes of CAZ/AVI in a specific patient cohort, demonstrating the therapeutic potential of this regimen for CRKP-induced CNS infections. This study contributes by expanding upon the previous research, specifically by including data on the optimization of CAZ/AVI dosing and its use in conjunction with local antibiotic administration. Additionally, while Zhao et al. (28) primarily concentrated on hospital-acquired CNS infections, this study extends those findings to a broader range of CRKP-induced CNS infections, demonstrating the feasibility and efficacy of this regimen in diverse clinical settings. This adds significant value by addressing a key gap in the literature regarding the role of local antibiotic therapy, such as intraventricular polymyxin B and amikacin, alongside systemic CAZ/AVI treatment.

Several of the patients involved in this study had undergone previous neurosurgical procedures, including hardware withdrawal and abscess drainage, which are critical for the management of CNS infections. These surgical interventions may play a significant role in controlling the infection, potentially confounding the impact of antibiotic therapy alone. It is important to consider the combined effects of surgical infection control and antimicrobial therapy when evaluating the outcomes in future studies. An important consideration in this study is the use of intrathecal colistin, aiming to achieve high local concentrations in the CSF and minimize systemic side effects. However, the potential contribution of intrathecal colistin to the overall clinical outcomes remains unclear. The observed improvements in the majority of patients may be partly attributed to the synergistic effect of intrathecal colistin in combination with CAZ/AVI. Future studies should consider evaluating the independent contribution of CAZ/AVI alone, as the use of intrathecal colistin could confound the results.

## Limitations

There are several limitations in this study, including the small sample size and the single-center design, which might limit the generalizability of the results. While this study provided valuable insights into the therapeutic potential of CAZ/AVI in treating CRKP-induced CNS infections, the small sample size might further restrict the external applicability of the findings. Therefore, the results should be interpreted with caution, and larger, multicenter studies are necessary to further establish the efficacy and safety of CAZ/AVI in this context. Additionally, the retrospective nature of the study indicates that the data collection could be influenced by incomplete or variable clinical documentation. Further studies with a larger, multicenter cohort and more detailed monitoring of CSF antibiotic concentrations will be beneficial in confirming the findings of this study.

## Conclusion

Despite the above-mentioned limitations, this study contributes valuable data to the growing body of literature on CAZ/AVI use in the treatment of CRKP-induced CNS infections. Given the rising prevalence of carbapenem-resistant pathogens, it is crucial to increase the reporting of real-world data on the use of CAZ/AVI to further refine treatment protocols and explore its potential role in combination therapies for multidrug-resistant infections.

In conclusion, the combination of intravenous CAZ/AVI with intracerebroventricular/intrathecal polymyxin B or amikacin proved to be a feasible and effective treatment for CRKP-induced CNS infections, resulting in an 88.9% cure rate in this cohort. The absence of major adverse effects indicates that this treatment regimen is safe when administered with close monitoring. However, CSF antibiotic concentrations need to be better documented to evaluate the true pharmacokinetics and effectiveness of CAZ/AVI in CNS infections. The early removal of intracranial implants is crucial to prevent treatment failure, particularly in cases of persistent or relapsing infections. Future studies should address the optimal duration of treatment, the role of local antibiotic administration, and further explore the CSF pharmacokinetics of CAZ/AVI to better tailor therapeutic strategies for CRKP-induced CNS infections. While this study supports the potential efficacy of CAZ/AVI for CRKP-induced CNS infections, the lack of clear new knowledge demonstrates the need for further research in this area. Future investigations should concentrate on evaluating CAZ/AVI as a monotherapy, optimizing dosing regimens based on pharmacokinetic and pharmacodynamic principles, and exploring its role in combination with other novel antibiotics. Additionally, studies should address the long-term outcomes and possible adverse effects associated with these therapies.

## Data Availability

The original contributions presented in the study are included in the article/supplementary material, further inquiries can be directed to the corresponding authors.
